# Contraceptive use and distribution of high-risk births in Nigeria: a sub-national analysis

**DOI:** 10.3402/gha.v8.29745

**Published:** 2015-11-09

**Authors:** Akanni Akinyemi, Sunday Adedini, Sennen Hounton, Ambrose Akinlo, Olanike Adedeji, Osarenti Adonri, Howard Friedman, Solomon Shiferaw, Abdoulaye Maïga, Agbessi Amouzou, Aluisio J. D. Barros

**Affiliations:** 1 Demography and Social Statistics Department, Obafemi Awolowo University, Ile Ife, Nigeria; 2 Demography and Population Studies Programme, University of Witwatersrand, Johannesburg, South Africa; 3 United Nations Population Fund, New York, NY, USA; 4 School of Research and Postgraduate Studies, Northwest University, Mafikeng, South Africa; 5 United Nations Population Fund, Abuja, Nigeria; 6 School of Public Health, Addis Ababa University, Addis Ababa, Ethiopia; 7 Institut Supérieur des Sciences de la Population, Ouagadougou University, Ouagadougou, Burkina Faso; 8 UNICEF, New York, NY, USA; 9 International Center for Equity in Health, Federal University of Pelotas, Capão do Leão, Brazil

**Keywords:** sub-national, estimates, contraceptive use, high-risk births, Nigeria

## Abstract

**Background:**

Family planning expansion has been identified as an impetus to harnessing Nigeria's demographic dividend. However, there is a need for data to address pockets of inequality and to better understand cultural and social factors affecting contraceptive use and health benefits. This paper contributes to addressing these needs by providing evidence on the trends and sub-national patterns of modern contraceptive prevalence in Nigeria and the association between contraceptive use and high-risk births in Nigeria.

**Design:**

The study utilised women's data from the last three Demographic and Health Surveys (2003, 2008, and 2013) in Nigeria. The analysis involved descriptive, bivariate, and multivariate analyses. The multivariate analyses were performed to examine the relationship between high-risk births and contraceptive use. Associations were examined using Poisson regression.

**Results:**

Findings showed that respondents in avoidable high-risk birth categories were less likely to use contraceptives compared to those at no risk [rate ratio 0.82, confidence interval: 0.76–0.89, *p*<0.001]. Education and wealth index consistently predicted significant differences in contraceptive use across the models.

**Conclusions:**

The results of this study suggest that women in the high-risk birth categories were significantly less likely to use a modern method of contraception relative to those categorised as having no risk. However, there are huge sub-national variations at regional and state levels in contraceptive prevalence and subsequent high-risk births. These results further strengthen evidence-based justification for increased investments in family planning programmes at the state and regional levels, particularly regions and states with high unmet needs for family planning.

**Paper context**

Using the last three Nigerian Demographic and Health Survey datasets, we examined the trends and sub-national patterns of modern contraceptive prevalence, as well as associations between contraceptive use and high-risk births in Nigeria. Findings showed that women in high-risk birth categories had lower uptake of modern contraceptive methods compared to those who were not in any high-risk birth category. The study further established huge sub-national variations in contraceptive prevalence and high-risk births, thus underscoring the need for increased investments in family planning programmes at the state and regional levels, particularly in areas with high unmet needs for family planning.

Nigeria is ranked among the 10 fastest growing populations in the world, is the most populous country in Africa, and the eighth most populous country in the world. The population is estimated to have grown from 56 million in 1970 to over 176 million in 2014 and is projected to be 184 million in 2015 with a growth rate of 3.2% (1). The total fertility rate (TFR) has been persistently high, from 5.7 in 2003 and 2008 to 5.5 in 2013, with regional estimates ranging from 4.3 in the South South region to 6.7 in the North West region. According to the 2013 Nigeria Demographic Health Survey (DHS), about 8.3% of births were unwanted and the adolescent birth rate was estimated at 122 per 1,000 women aged 15–19 years (only half the rate of women 25–29 years), with unmet need for contraception currently estimated at almost 50% among married women (2). These present significant challenges to harnessing the demographic dividend and creating sustainable development.

Although there has been a small increase in modern contraceptive use, from 8.9% in 2003 to 10.5% in 2008 and 11.1% in 2013 at the national level, the rate is still very low compared to the level of modern contraceptive prevalence in many other developing nations, which can be as high as 70% (3). Contraceptive use, particularly modern contraceptives, remains the main proximate determinant of fertility. According to the 2013 DHS, overall contraceptive prevalence among married women in Nigeria was estimated at 15.1%. However, there were wide variations at the sub-national level. Previous studies and the DHS documented reasons for non-use of contraceptive methods (2, 4–6), which included demographic and socioeconomic factors, fertility-related factors, opposition by partner, lack of knowledge, and family planning method–related reasons (2, 4–6). The need to improve access to and voluntary use of contraceptive methods is considered as a very important priority, particularly in a bid to harness the potential demographic dividends and improve the quality of life of individuals and the well-being of families.

Research has shown the benefit of expanding family planning services for improving maternal and child health, as well as increasing the well-being of individuals, families, and communities (7–12). Family planning promotion has been identified as a very important factor in addressing high-risk births and three major streams of preventable child and maternal deaths and in creating an AIDS-free generation (13). Research has shown that there is an inverse relationship between contraceptive use and high-risk births and that high-risk births are directly related to maternal and child mortality rates (8, 14–17). The estimates of high-risk births in Nigeria according to the 2013 DHS showed that two-fifths of all births to women and 32% of births to currently married women are high-risk births. About 23% of births to all women and 48% of all births to currently married women are categorised as multiple-risk births (2).

However, for a country like Nigeria with heterogeneous configurations, there is a need for a broader understanding of family planning and reproductive health issues, taking into account particular sectorial and sub-national differences. Even within one state, qualitative evidence confirmed variations in family planning adoption across local districts and communities in Nigeria (6). In addition, there is a need for evidence-based policy by demonstrating, at the state level, potential gains to maternal and child health associated with increasing contraceptive use. There are wide regional- and state-level variations in family planning and reproductive health indicators in Nigeria; providing a trend analysis at regional and state levels of the nexus between family planning and distribution of high-risk births may provide information for family planning intervention programmes that will respond to these diverse contexts.

This paper assesses the linkage between contraceptive prevalence, fertility levels and trends, and distribution of high-risk births in Nigeria at the sub-national level and uses the results to formulate recommendations for policy and programming. Sub-national divisions in Nigeria by region and state are shown in Fig. 1.

**Fig. 1:**
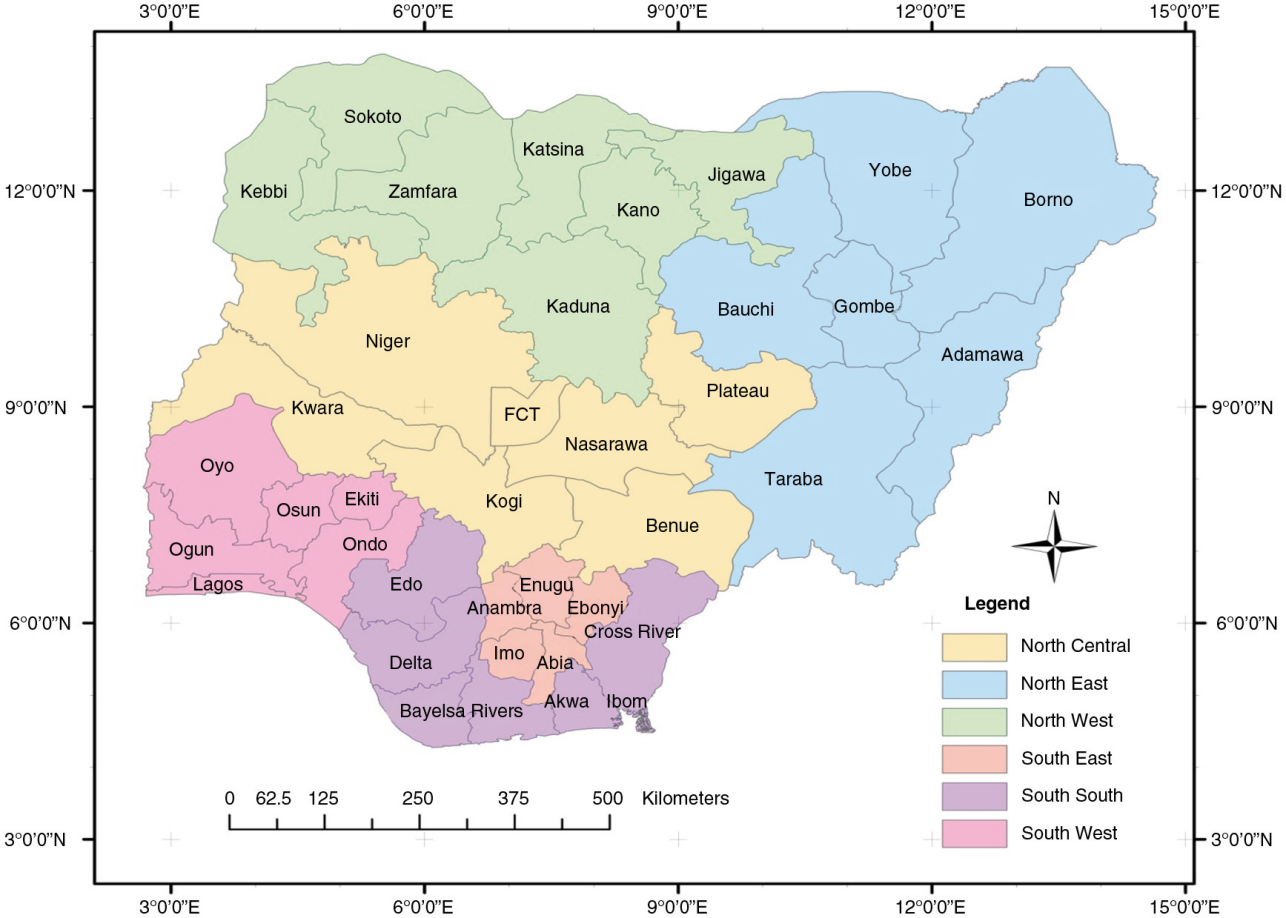
Map of Nigeria, showing regions and states.

## Methods and Data

We utilised datasets from the last three DHSs in Nigeria (2003, 2008, and 2013) (2, 4, 18). Each of these surveys employed a cross-sectional design to collect nationally representative data from women of reproductive age (15–49 years). The indicators used include the following: high-risk births, modern contraceptive prevalence rate (mCPR), TFR, infant mortality rate, birth interval, and mother's age. Apart from the descriptive analysis, bivariate and multivariate analyses were performed to examine the influence of high-risk births on the outcome measure (contraceptive use). At the multivariate level of analysis, using only the 2013 DHS, Poisson regression was used to model the relationship between modern contraceptive use and high-risk births, controlling for education, wealth index, and region of residence. Wealth index is a proxy measure for standard of living because information on incomes is subjective and unreliable. Principal component analysis was used to generate wealth index from information based on each household's ownership of consumer goods, dwelling characteristics, and other household characteristics. Multivariate results were presented as rate ratios and respective 95% confidence intervals (CIs). Our outcome variable (modern contraception, meaning current use of at least one modern method of contraception at the time of the survey by married women or those in a union, aged 15–49 years) is a dichotomous variable. Nonetheless, preference was given to Poisson regression over binary logistic regression for this analysis, as many studies have argued and shown that Poisson regression is a better alternative for the analysis of cross-sectional studies with binary outcomes than logistic regression (19–21).

The main predictor variable was high-risk births. The births were categorised as 1) no risk, 2) unavoidable high-risk, and 3) avoidable high-risk. Births in the category of ‘no risk’ were second or third births to mothers between the ages of 18–34 years and with a preceding birth interval not less than 24 months, strictly representing lowest possible risk. ‘Unavoidable high-risk’ births were first births to mothers between the ages of 18–34 years (2). ‘Avoidable high-risk’ births were those to women aged 17 or younger, to women aged 35 or older, with a preceding birth interval of less than 24 months, and/or parity of four or higher (2). The high-risk birth variable was generated from the birth recode dataset to ensure that all births to interviewed women in the last 5 years were captured in the computation of high-risk births. This dataset was then merged with the women's individual recode dataset, since women are the main unit of analysis. The merged dataset thus excludes women who had not given birth in the last 5 years preceding the survey. Other explanatory variables selected include education, household wealth status, and region of residence. The main outcome variable was the use of modern contraception; we adopted the DHS categorisation of modern methods. Contraceptive use was categorised as 1) currently using modern method or 2) not currently using modern method. These variables have been reported as important factors influencing child survival (22, 23).

All analyses were performed using Stata 13 (24), taking into account the complex design of the DHS surveys.

## Results

### Fertility trends and dynamics

**Fig. 2:**
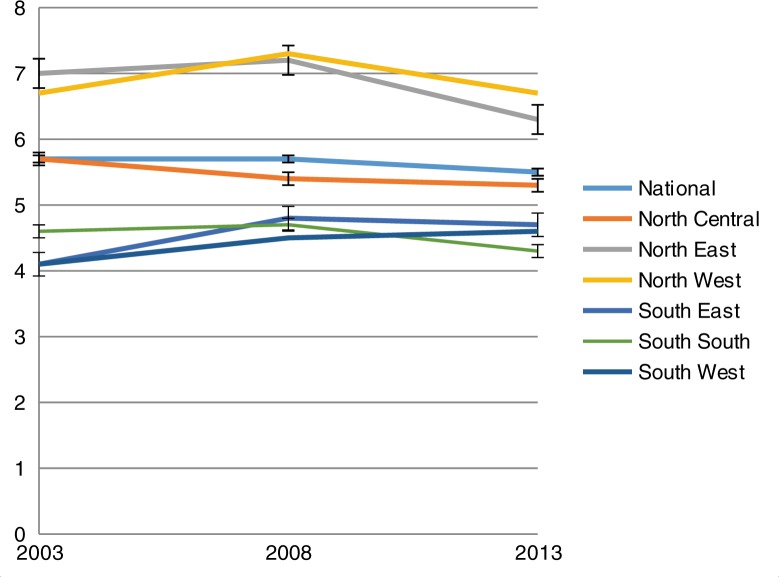
Fertility trends, national and regional estimates.

The national estimate of TFR showed a minimal decline from 5.7 in the periods 2003 and 2008 to 5.5 in 2013. Across the six regions as presented in Fig. 2, there are noticeable variations in trends within this period. Although the rates across the years remained highest in the North East and North West regions, there was a slight decline in the rates in the two regions between 2008 and 2013, from 7.2 to 6.3 in the North East region and 7.3 to 6.7 in the North West region. Except for the North Central region, there was a slight increase in the rates across the regions between 2003 and 2008. In addition, with the exception of only the South West region, there was a general decline between the 2008 and 2013 estimates across the region.

**Fig. 3:**
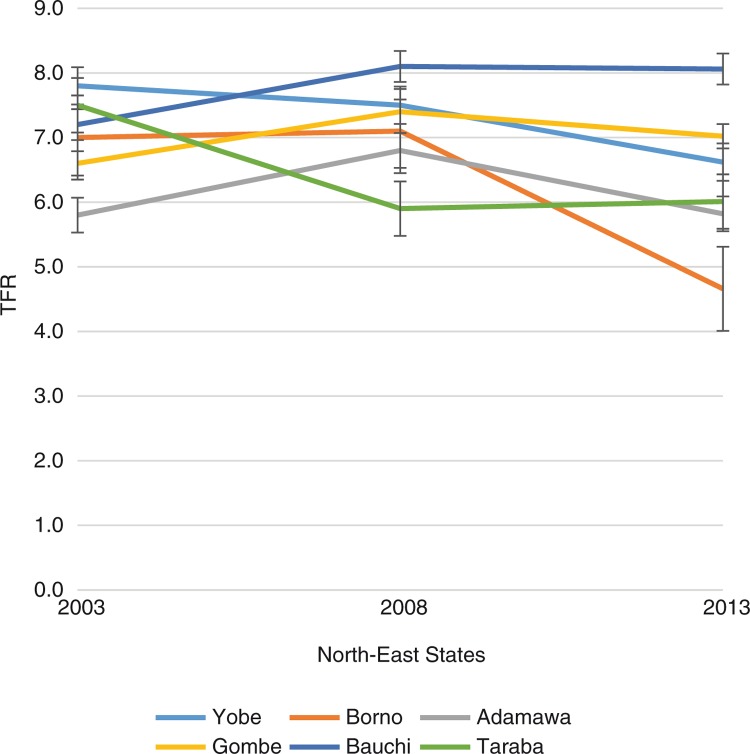
Fertility trends across the states in North East Nigeria, 2003–2013.

However, within the regions, the states exhibited wide variations. For instance, in the North East region, comprised of six states, there were wide variations in the TFR as well as the trend over time. Three states showed a decline in TFR between 2003 and 2013 while two of the states showed an increase. In Bauchi State, for instance, the TFR increased from 7.2 in 2003 to 8.1 in 2013, whereas in Borno State, there was a decline from 7.0 in 2003 to 4.7 in 2013. In 2003, the range of TFR across the states was 7.8 in Yobe compared with 5.8 in Adamawa; it was 8.1 in Bauchi and 5.9 in Taraba in 2008; and 8.1 in Bauchi and 4.7 in Borno in the year 2013. Figure 3 presents the details.

**Fig. 4:**
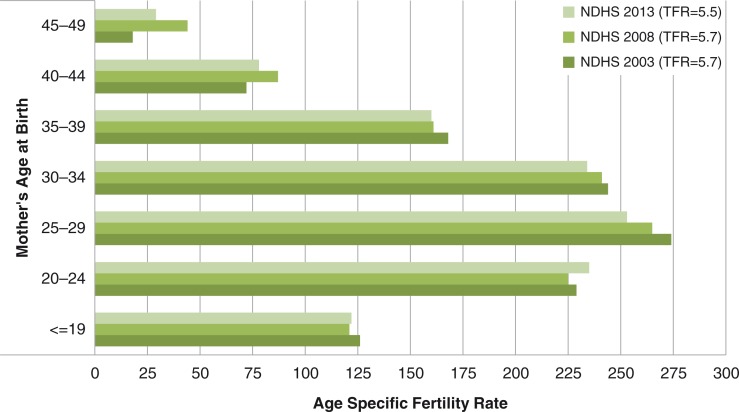
Trends in age-specific fertility rate.

The age-specific fertility rates (ASFRs) presented in Fig. 4 show that across the three surveys, the ASFR was about 125 births per thousand young women below 20 years, while the number of births per thousand women aged 40–44 and 45–49 increased from 50 in year 2003 to 60 in 2008 and soared to about 80 in 2013. The ASFRs among women aged 20–24 and 45–49 have increased over the 10-year period and this rise portends serious consequences for the profile of high-risk births in Nigeria.

**Fig. 5:**
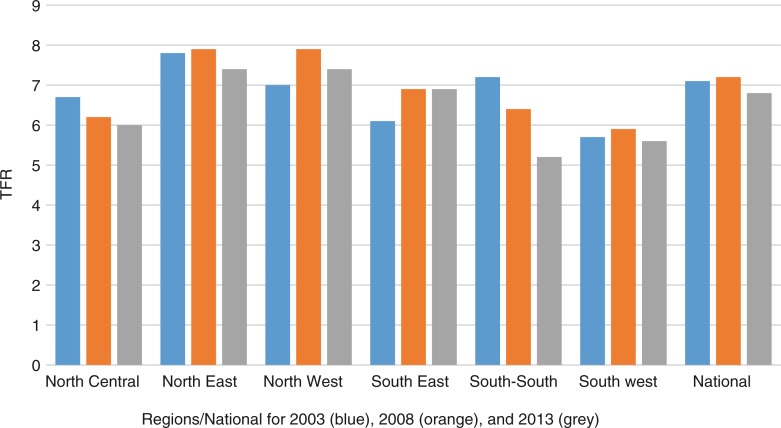
Sub-national trends in total fertility rate for women who had their first birth before 18 years.

**Fig. 6:**
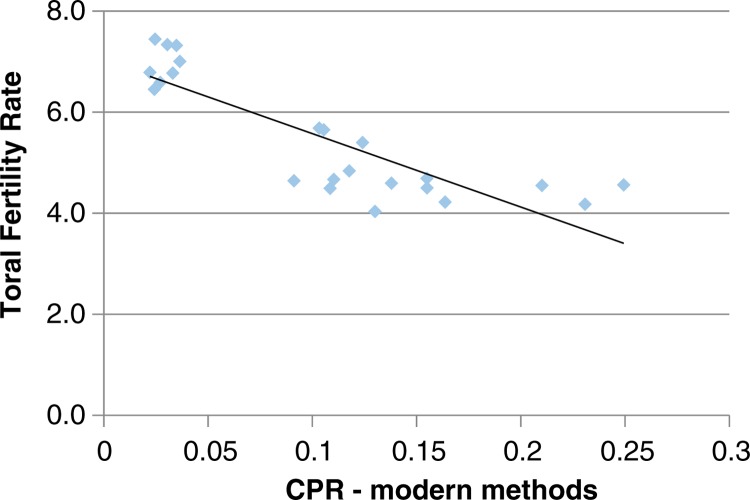
Relationship between total fertility rate and modern contraceptive prevalence rate in Nigeria.

The age at which women begin childbearing may influence other fertility-related variables. Nationally, the mean age of first birth across the surveys was 18 years. However, this varies from 17 years in the North West to 22 years in the South East and South West regions. The TFR for women who started childbearing early (aged 18 years or below) is presented in Fig. 5. In the North West and North East regions, women who had their first birth before 18 years had a TFR of between 7 and 8 children, compared with 5.3 in the South West and 6.8 in the South East region. Figure 5 shows the relationship between TFR and mCPR across the regions; the distribution shows an inverse relationship between TFR and mCPR. Regions with considerably lower mCPR have very high fertility levels, ranging from 6.2 to 7.5 as shown in Fig. 6.

### Trends and regional distribution of high-risk births by fertility and contraception use

**Fig. 7:**
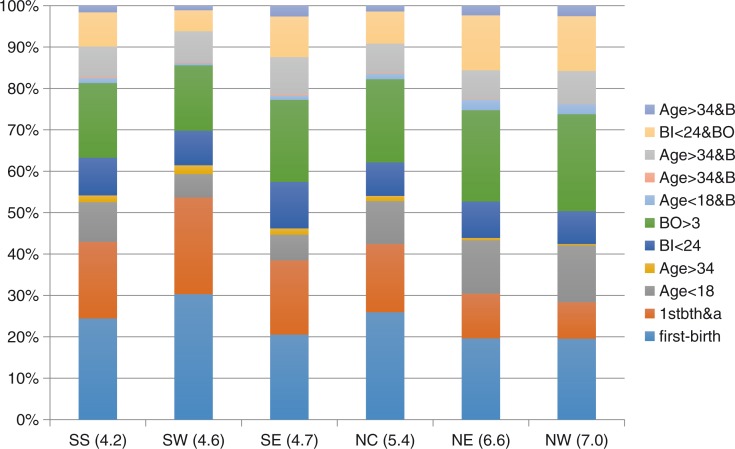
Distribution of high-risk births by region, Nigeria 2013.

The distribution of births by risk factors varies considerably across the regions and by the level of TFR in each region. Figure 7 shows the distribution of births by risk factors and regional estimates of TFR. The proportion of births associated with avoidable risk is higher in the North West (72%) and North East (69%) regions compared with the South South (57%) and South West (45%) regions. The proportion of avoidable risk for all births is higher in regions with relatively higher levels of TFR (North West and North East) compared with the two regions with relatively lower levels of TFR (South West and South South). For the two regions with the highest level of TFR (North West and North East), more than 12% of births were to women less than 18 years, whereas only about 4% of births in the South West region and 7% of births in the South South region were among young women 18 years or less. The two regions (North West and North East) with the highest level of TFR have 15% of multiple risk factors (closely spaced birth interval, high birth order, and age of mothers) compared with 8% in South South Nigeria and 5% in South West Nigeria.

The proportion of women with high birth order was higher in North West and North East Nigeria, which constituted over 20% of all births in those regions, compared with less than 15% of all births in the South West and South South regions. South East Nigeria, however, had the highest proportion of closely spaced births, which constituted almost one-tenth of all births in the region, compared with about 6% of all births in North West Nigeria.

Further analysis to examine the patterns and trends of the distribution of births by risk factors in two regions (South South, with the lowest TFR, and North West, with the highest TFR) was also done (analysis not shown). In the South South region, the general pattern of the distribution of high-risk births shows that between 15 and 25% of all births across the states in three surveys are high parity. Bayelsa State, with the highest TFR in the year 2003, had the highest proportion of avoidable high-risk births, estimated at 65% in 2003, 63% in 2008, and 60% in 2013. However, there was no regular pattern in distribution of high-risk births and fertility levels in the states across the three surveys. In North West Nigeria, however, a major spotlight is on Kaduna State with a sharp decline in TFR from 7.3 in 2003 to 4.1 in 2013 and a corresponding decline in the proportion of high-risk births considered avoidable, which also dropped from 67% in 2003 to 60% in 2013. In contrast, Sokoto State experienced an upward trend in TFR from 5.2 in 2003 to 7.0 in 2013. The proportion of high-risk births categorised as avoidable also increased from 69% in 2003 to 74% in 2013. Generally, across the states in North West Nigeria, high-parity births constituted between 20 and 30% of all high-risk births, except for Sokoto in 2008. In addition, about 15–20% of all births across the states, except Sokoto in 2008, were among young women less than 18 years.

**Fig. 8:**
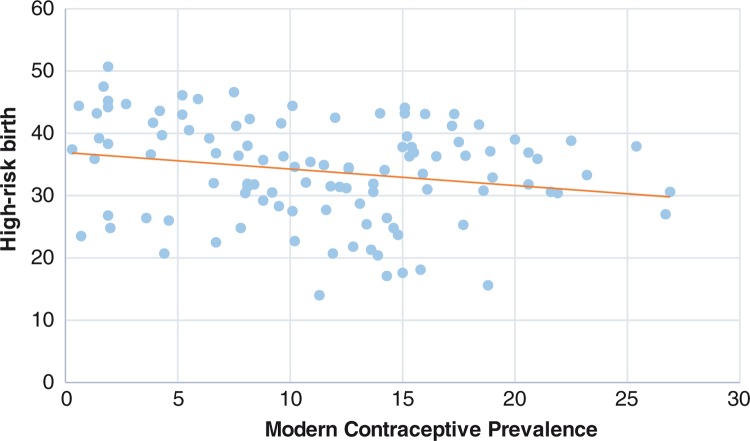
Distribution of proportion of high-risk birth by modern contraceptive prevalence, for regions (blue) and states (orange), 2003–2013.

Figure 8 presents the distribution of high-risk births by modern contraceptive prevalence for all the surveys across regions and states. The graph shows the spread of the dots that reflects an inverse relationship between mCPR and the distribution of high-risk births. The graph shows that the proportion of births in high-risk categories is relatively higher in regions/states with lower levels of modern contraceptive prevalence.

### Multivariate analysis

Using Poisson regression, the relative risks (adjusted and unadjusted) of using a modern method of contraception are presented in Table 1. The results show that relative to women who were not in any high-risk birth category, the odds of using a modern method of contraception were significantly higher for women whose last births were unavoidable high-risk births (rate ratio 0.82, CI: 0.76–0.89, *p*<0.001). Adjusting for education and wealth index in Model 1, as shown in Table 1, results indicated that women in avoidable risk categories had a slightly higher rate of modern contraceptive use (rate ratio: 1.13, CI: 1.05–1.22, *p*<0.01) compared to those at no risk. In addition, rates of contraceptive use increased with higher levels of education and wealth status. For instance, results (Model 1) showed that women from the richest households (rate ratio 8.43, CI: 6.52–10.90, *p*<0.001) and women who had higher education (rate ratio 6.02, CI: 5.09–7.13, *p*<0.001) were more likely to use contraception than those in the reference categories. Adjusting for region of residence (Model 2) did not considerably alter these results. The relationship between region of residence and contraceptive use was significant, as results (Model 2) revealed significantly lower rates of contraceptive use in all of the other five regions compared to the South West region, the reference category.

**Table 1:** Poisson regression analysis showing rate ratios for modern contraceptive use in relation to high-risk births (2013 NDHS)

Characteristics	Unadjusted model	Adjusted model (Model 1)	Adjusted model (Model 2)
High-risk birth			
No risk	1	1	1
Unavoidable high risk	1.10 (0.99–1.23)	0.81 (0.73–0.90)***	0.81 (0.73–0.99)***
Avoidable high risk	0.82 (0.76–0.89)***	1.13 (1.05–1.22)**	1.20 (1.11–1.29)***
Wealth quintiles			
Poorest	1	1	1
Poorer	3.81 (2.96–4.90)***	2.59 (2.00–3.34)***	2.17 (1.68–2.81)***
Middle	9.19 (7.23–11.68)***	4.25 (3.30–5.48)***	3.06 (2.37–3.95)***
Richer	15.18 (12.00–19.20)***	5.59 (4.33–7.22)***	3.86 (2.99–5.00)***
Richest	27.7 (22.00–34.91)***	8.43 (6.52–10.90)***	5.54 (4.27–7.18)***
Education			
None	1	1	1
Primary	6.67 (5.83–7.64)***	4.10 (3.54–4.75)***	2.98 (2.55–3.49)***
Secondary	10.87 (9.58–12.33)***	5.01 (4.31–5.82)***	3.61 (3.08–4.24)***
Higher	16.77 (14.60–19.26)***	6.02 (5.09–7.13)***	4.25 (3.56–5.08)***
Region			
South West			1
North Central			0.90 (0.83–0.99)*
North East			0.37 (0.32–0.43)***
North West			0.33 (0.28–0.38)***
South East			0.67 (0.60–0.75)***
South South			0.82 (0.75–0.89)***

*** Significant at 0.05;

** significant at 0.01;

* significant at 0.001.

## Discussion

Expanding family planning programmes has been identified as a major prerequisite in order to harness the benefits of the demographic dividend in Nigeria (25–27). Part of realising this dividend requires a significant reduction in fertility as well as unrestricted access to voluntary and safe contraception through an effective family planning programme. Understanding the needs of individuals as well as sub-national variations in family planning promotion in Nigeria has been identified as a major gap in the low uptake of family planning.

In a bid to connect the present analyses with sub-national information at the state level, a review of information showed that the variations presented were confirmed and supported by evidence across the states. For instance, in the South South and South East regions, there are states whose fertility and family planning indicators are similar to those in the North West region, and this presents a wide dispersion from the regional estimates. Bayelsa and Ebonyi states are two examples of states in South South and South East Nigeria where family planning and maternal indices are relatively poor and similar to those in northern Nigeria. The geographic terrain of Bayelsa State, for instance, makes family planning and maternal health interventions challenging because about 90% of the communities are located in hard-to-reach areas in the riverine, core waterlogged Niger Delta. This is compounded by the religious beliefs of the people and general apathy towards modern family planning methods. In addition, the differentials in fertility, mCPR, and high-risk births are more pronounced in regions like the South East and South South. As a result of this, much is required in terms of investments in family planning programmes in such states as Bayelsa, Ebonyi, and others in Nigeria where contraceptive prevalence is low and high-risk births, as well as childhood and maternal mortality, remain high.

Further, Kaduna State in the North Central region of Nigeria showed family planning and maternal health indicators levels similar to those in South West Nigeria – the region with, relatively, the best reproductive health indicators in Nigeria. We also noted (analysis not shown) that Kaduna State, over the years, experienced a drastic drop in fertility rates. This decrease is perhaps partly due to the intensive efforts by the State Ministry of Health in collaboration with the National Urban Reproductive Health Initiative and United Nations Population Fund, which adopted an evidence-based approach to programming interventions.

The evidence of improvement of family planning and maternal health indicators in Kaduna State further illustrates the importance of state interventions in programmes and policies targeted towards expanding family planning outreach. This process requires a significant investment in positive directions including those targeted towards women's empowerment through education and direct investment in healthcare. This could be a good approach for more state investments in health and education.

The TFR of 5.5 at the national level and of over 7 in some regions is unacceptably high and thus may hinder reaping the gains of the demographic dividend. It is apparent that improving provision of family planning supplies and services, and the expansion of education and job opportunities for girls and women (25), have direct potential for improving quality of life by reducing the burden of high-risk births. The policy issues from this analysis further reiterate that expanding family planning is important in improving maternal and child health. There is a need to improve funding by state governments and stakeholders in expanding the reach of family planning services.

## Conclusions

As established in this study, expanding family planning services will undoubtedly lead to reduction in the burden of high-risk births in the various regions and states of Nigeria. Currently, there are huge state and regional differentials in the supply and demand for modern contraception. In addition, fertility remains significantly high and mCPR remains considerably low in many states across the country. Worse still, high-risk births remain very high in many states and regions. Hence, the sub-national analyses presented in this paper suggest that the demographic dividend possibility remains a mirage in many areas and regions of the country. There is therefore the need for sub-national investment and policies to improve family planning services, taking into account regional- and state-level particularities. Such policies and programmes need to recognise socio-cultural variations in the country.
